# Low prevalence of human mammary tumor virus (HMTV) in breast cancer patients from Myanmar

**DOI:** 10.1186/s13027-017-0130-0

**Published:** 2017-04-12

**Authors:** Thar Htet San, Masayoshi Fujisawa, Soichiro Fushimi, Teizo Yoshimura, Toshiaki Ohara, Lamin Soe, Ngu Wah Min, Ohnmar Kyaw, Xu Yang, Akihiro Matsukawa

**Affiliations:** 1 Department of Pathology and Experimental Medicine, Graduate School of Medicine, Dentistry and Pharmaceutical Sciences, Okayama University, 2-5-1 Shikata, Okayama, 700-8558 Japan; 2 Department of Pathology, Himeji Red Cross Hospital, Himeji, Japan; 3 Department of Pathology, Myeik General Hospital, Myeik, Myanmar; 4 Department of Pathology, Sakura Specialist Hospital, Yangon, Myanmar; 5 Immunology Research Division, Department of Medical Research, Yangon, Myanmar

**Keywords:** Human mammary tumor virus, Mouse mammary tumor virus, Breast cancer

## Abstract

**Background:**

Human mammary tumor virus (HMTV) is 90–95% homologous to mouse mammary tumor virus (MMTV), one of the causal agents of murine mammary tumors. HMTV (MMTV-like) sequences were reported to be present in human breast cancers from several populations with a prevalence range of 0–78%; however, the prevalence of HMTV in breast cancers from Myanmar remains unknown.

**Methods:**

Fifty-eight breast cancer samples from Myanmar women were examined in this study. DNA was isolated from formalin-fixed paraffin-embedded specimens, and HMTV envelope sequences were detected by semi-nested PCR. The sequence of the PCR products was also confirmed.

**Results:**

Only 1.7% (1 of 58) of the breast cancers were positive for HMTV, and the sequence of PCR products was 98.9% identical to the reference HMTV sequence (GenBank accession No. AF243039). The tumor with HMTV was grade III invasive ductal carcinoma, 7.0 cm in size with lymph node metastasis (T3, N1, M0).

**Conclusions:**

We, for the first time, investigated the presence of HMTV in Myanmar breast cancer patients. In accordance with other Asian studies, the prevalence of HMTV in Myanmar was quite low, supporting the hypothesis that Asian breast cancers have different etiologies than in Western countries, where HMTV is more prevalent.

## Background

Worldwide, breast cancer is the most frequently diagnosed cancer affecting women, with an estimated 1.7 million cases and 521,900 deaths in 2012 [1, 2]. The incidence of breast cancer is higher in developed countries, while the mortality is higher in developing countries. These discrepancies in incidence and mortality are attributed to early detection as well as risk factors including geographic variation, racial/ethnic background, genetic variation, lifestyle, and reproductive patterns associated with urbanization and economic development [3, 4].

The etiology of human breast cancer can be significantly affected by environmental factors, including viruses [5, 6]. Among them, mouse mammary tumor virus (MMTV) is a non-acute transforming type B retrovirus that causes the majority of mammary tumors in mice. MMTV induces premalignant lesions and malignant tumors of the breast by acting as an insertional mutagen or activating the transcription of nearby oncogenes [7, 8]. In 1995, retroviral sequences 90–95% homologous to MMTV were detected in 39% of human breast cancers in the United States [9]. Subsequently, a 9.9-kb proviral structure, which was 95% homologous to MMTV, was successfully amplified from two distinct human breast cancers. The retrovirus with MMTV-like sequence was subsequently designated human mammary tumor virus (HMTV) [10]. Very recently, MMTV-like sequences were found in breast tissues prior to the development of virus-positive breast cancer, indicating a possible causal role in the development of breast cancer [11]. To understand the involvement of HMTV/MMTV-like sequence in the carcinogenesis of breast cancer, it is important to obtain more clinical and epidemiological data in breast cancer worldwide.

As with other countries, breast cancer is a leading cause of morbidity and mortality in Myanmar women [12]. The purpose of this study was to investigate the prevalence of HMTV in breast cancers in Myanmar.

## Methods

### Study subjects

In this study, we employed 58 breast cancer cases diagnosed in 2015 at Myeik General Hospital (Myeik City, Myanmar) and Sakura Specialist Hospital (Yangon City, Myanmar). All hematoxylin and eosin-stained sections were reviewed by two independent pathologists. The criteria defined by the World Health Organization (2012) were used for the histopathological diagnosis and classification of breast carcinoma [13]. Nottingham combined histological grading system [14] was used for tumor grading. American Joint Committee on Cancer staging system 8^th^ edition was applied for tumor staging. The experimental protocol employed in this study was approved by the Ethics Committee of Okayama University and the Ethics Review Committee of Department of Medical Research of Yangon City (Myanmar).

### DNA extraction

Two to four 10-μm-thick sections were cut from each paraffin block, and genomic DNA was extracted using the Nucleospin DNA FFPE XS kit (Macherey-Nagel, Düren, Germany) according to the manufacturer’s instructions. The amount of extracted DNA was measured on a Nanodrop 1000 spectrophotometer (Thermo Fisher Scientific, Waltham, MA, USA). The A260:A280 ratio was used to measure the purity of DNA (~1.80). DNA quality was also confirmed by PCR amplification of the 268 bp β-globin gene using GH20 and PC04 primers (Table 1). All samples employed in this study were qualified for HMTV detection.

**Table 1:** List of primers, their sequences and positions in the genome

gene	primer	sequence (5´–3´)	nucleotide position
β-globin	GH20	GAAGAGCCAAGGACAGGTAC	1417-1436^a^
	PC04	CAACTTCATCCACGTTCACC	1684-1665^a^
HMTV	5 F	GTATGAAGCAGGATGGGTAGA	235-255^b^
	MR1	CCTCTTTTCTCTATATCTATTAGCTGAGGTAATC	480-446^b^
	2NR	GTAACACAGGCAGATGTAGG	423-404^b^
mt DNA	mt15982F	AGACGCACCTACGGTGAAGA	15982-16001^c^
	mt16115F	TGCCAAACCCCAAAAACACT	16115-16134^c^
	mt16267R	AGAGTTTTGGTTCACGGAACATGA	16267-16244^c^

^a^Refers to *Homo sapiens* β-globin gene, complete cds (Genbank AH001475)

^b^Refers to human mammary tumor virus SAG pseudogene, complete sequence (Genbank AF243039)

^c^Refers to *Mus musculus* complete mitochondrial genome, strain Balb/cJ (Genbank AJ512208)

### Detection of HMTV sequence by PCR

The detection of HMTV DNA sequences was performed using a semi-nested PCR approach. Primers were carefully selected from those stated to successfully amplify HMTV sequences in previous literature [15–18]. In the first-round of PCR, primers 5 F and MR1 were used to amplify a 246-bp segment. In the second-round of PCR, the same forward primer (5 F) and a different reverse primer (2NR) were used to amplify a 189-bp HMTV sequence. PCR reactions were performed in a 20 μl volume and contained 0.02 μM and 0.2 μM of primers, together with reagents from the Pyromark PCR kit (QIAGEN, Hilden, Germany) according to the manufacturer’s instructions. Approximately 250 ng of extracted DNA was used as template for first-round PCR and 2 μl of first-round PCR product was used as template for second-round PCR. PCR products were electrophoresed on 2% agarose gels containing ethidium bromide and visualized with ultraviolet light. RCB0526:Jyg-MC(A) cells (Riken Bioresource Center, Tsukuba, Japan), a murine mammary tumor cell line expressing high MMTV levels, were cultivated, harvested and the extracted DNA was used as a positive control.

To exclude murine DNA contamination in the HMTV-positive sample, mouse-specific mitochondrial (mt) DNA was amplified by semi-nested PCR using the primers mt15982F and mt16267R for the first-round PCR and mt16115F and mt16267R for the second-round PCR, which yielded a final PCR product of 153-bp. DNA extracted from the 4 T1 murine breast cancer cell line was used as positive control.

The list of all primers used and their positions in the genome are shown in Table 1. PCR conditions were as follows: β-globin: 95 °C for 15 min, 40 cycles of 95 °C for 30 s, 55 °C for 30 s, and 72 °C for 1 min, and then 72 °C for 7 min; HMTV: first-round PCR: 95 °C for 15 min and 40 cycles of 30 s at 95 °C, 30 s at 58 °C and 1 min at 72 °C, and then 72 °C for 7 min; second-round PCR: 95 °C for 15 min and 35 cycles of 30 s at 95 °C, 30 s at 60 °C and 1 min at 72 °C, and then 72 °C for 7 min; mt DNA: first-round PCR: 95 °C for 15 min and 40 cycles of 30 s at 95 °C, 30 s at 55 °C and 1 min at 72 °C, and then 72 °C for 7 min; second-round PCR: 95 °C for 15 min and 35 cycles of 30 s at 95 °C, 30 s at 55 °C and 1 min at 72 °C, and then 72 °C for 7 min.

### DNA sequencing

Sequencing was performed using an ABI3130*xl* Genetic Analyzer (Applied Biosystems, Waltham, MA, USA) at the Central Research Laboratory of Okayama University. PCR products were isolated from gels, sequenced and aligned by a BLAST search (NCBI).

## Results

### Patient characteristics

Clinical data for the 58 breast cancer patients are shown in Table 2. Ages ranged from 30 to 81 years with a mean age of 50.3 years. Tumor sizes ranged from 1.5 cm to 7.2 cm with an average size of 4.0 cm. Most of the cancers (97%) were invasive ductal carcinoma with high histological grade (grade II and III). No grade I malignancies were found. There were lymph nodes metastases in 57% of the cases.

**Table 2:** Clinical data for the enrolled breast cancer patients

	categories	number of cases (%)
Age (years)	<35	4 (6.9)
	35–50	24 (41.4)
	>50	30 (51.7)
Tumor size (cm)	≦2.0	4 (6.9)
	2.1–5.0	39 (67.2)
	>5.0	15 (25.9)
Pathological diagnosis	Invasive ductal carcinoma	56 (96.6)
	Mucinous carcinoma	1 (1.7)
	Carcinoma with neuroendocrine differentiation	1 (1.7)
Histological grade	Grade I	0 (0.0)
	Grade II	26 (44.8)
	Grade III	32 (55.2)
Lymph node metastasis	Absent	25 (43.1)
	Present	33 (56.9)
Stage	Stage I	4 (6.9)
	Stage II	30 (51.7)
	Stage III	22 (37.9)
	Stage IV	2 (3.4)

### Detection of HMTV

**Fig. 1:**
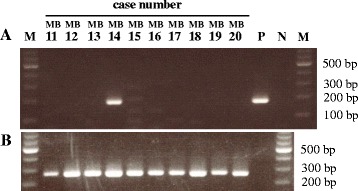
Gel electrophoresis of PCR products. **a**. HMTV Semi-nested PCR was performed using genomic DNA purified from each paraffin-block. Case numbers from MB11 to MB20 are shown. M, 100-bp DNA ladder; P, positive control; N, negative control. **b**. β-globin PCR was performed using the same DNA as above

**Fig. 2:**
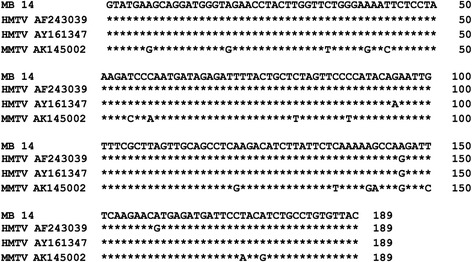
Sequence analysis of the PCR product. Sequence of the PCR product from MB14 is aligned with reference HMTV sequence (AF243039), HMTV sequence detected in a Vietnamese woman (AY161347) and reference sequence of the MMTV control (AK145002) retrieved from GenBank. Stars show the conserved sites along the alignment

**Fig. 3:**
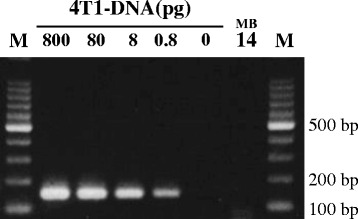
Exclusion of murine DNA contamination in MB14-DNA. Murine mitochondrial DNA semi-nested PCR was performed using MB14-DNA (250 ng). Serially diluted DNAs from murine 4 T1 cells were used as positive controls. M, 100-bp DNA ladder

Experiments were conducted to analyze the prevalence of HMTV. Genomic DNA was extracted from each paraffin block and its quality was measured using the A260:A280 ratio (>1.80) and a distinct β-globin PCR product. HMTV sequence was investigated in all 58 samples using semi-nested PCR, which revealed one case (MB14) was positive for HMTV (Fig. 1). The PCR reaction was confirmed by repeating the semi-nested PCR using newly extracted DNA from the paraffin block (not shown). The positive band was cut from the gel, and the PCR product was sequenced. The complete 189-bp PCR product sequence was aligned to two published HMTV sequences, and the reference sequence of the positive control (Fig. 2). The sequence was 98.9% identical to the original proviral HMTV sequence (GenBank AF243039) and HMTV sequence from Vietnam (GenBank AY161347). Although MB14-sequence showed 92% homology with control MMTV (GenBank AK145002), we attempted to exclude murine DNA contamination in MB14-DNA. For this, MB14-DNA (250 ng) was amplified using primers for mouse mitochondrial DNA. 4 T1-DNA was used as a positive control murine DNA. The data in Fig. 3 demonstrated that there was no contamination of murine DNA in MB14-DNA. The detection limit of this assay was more than 0.8 pg DNA (Fig. 3). The HMTV-positive case was invasive carcinoma, 7.0 cm in size, histological grade III, with lymph node metastasis.

## Discussion

**Fig. 4:**
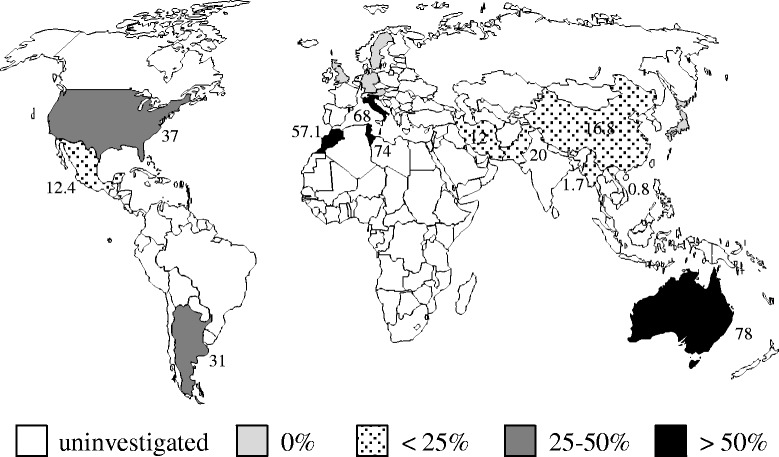
The prevalence of HMTV in breast cancers from 17 countries. The percent prevalence in each country is shown on the map except 0 prevalence

HMTV has been detected at different frequencies in different countries. We, for the first time in this study, investigated the prevalence of HMTV in breast cancers in Myanmar. Semi-nested PCR and sequencing data showed that the prevalence of HMTV was very low (1.7%, 1 of 58 cases). To the best of our knowledge, the prevalence of HMTV (MMTV-like) sequences in breast cancers has been reported for 17 countries, including the present study. Figure 4 shows the prevalence of HMTV on the world-map from these reports.

The prevalence of HMTV shows geographic heterogeneity. A high prevalence of HMTV was detected in North and South America, Australia and Mediterranean countries, where the range is from 12 to 78% (average 49.4%) [18–25]. Conversely, HMTV was not detected in Central and Northern Europe [26–29]. In Asia, no or few cases are positive for HMTV in Japan [30], Iran [31], Vietnam [15] and Myanmar (this study). The prevalence of HMTV in Myanmar breast cancers (1.7%) is comparable to that of a Vietnamese study (0.8%) [15]. More intriguingly, the sequence of HMTV reported in the Vietnamese study (GenBank AY161347) was 98.9% identical to that seen in this Myanmar case, suggesting there may be a close etiological relationship between the two countries. Although the prevalence in China and Pakistan was high (16.8 and 20.0%, respectively) [32, 33], no sequence was confirmed in the Chinese study, and only 2 of 16 positive cases were confirmed in the Pakistani study. A recent study in Iran showed that 12% of cases were positive, but none of the sequences were confirmed [34]. These data appear to be contingent and necessitate further study.

Interestingly, Asian countries with zero or low HMTV prevalence such as Japan, Vietnam and Myanmar have low breast cancer incidence (51.5, 23.0 and 22.1 per 100,000 women, respectively) compared with countries with high HMTV prevalence like the United States, Italy and Australia (92.9, 91.3 and 86.0 per 100,000 women, respectively) (http://globocan.iarc.fr.). Breast cancer develops at earlier ages in Asia (peak incidence between 40 and 50 years) than in Western countries (peak incidence between 60 and 70 years) [35]. These differences may reflect distinct characteristics and etiological backgrounds. There is an interesting report hypothesizing that human breast cancer could be correlated with the natural ranges of different species of wild mice [36]. *Mus domesticus,* having the highest numbers of endogenous MMTV proviral loci, inhabits the United States, Italy and Australia, where the prevalence of HMTV in breast cancers is high. On the other hand, *M. musculus* and *M. castaneus*, having the fewest MMTV proviral loci, ranges from central Europe east to China and Japan and from southern China to central Iran, respectively.

A question arises about the sensitivity of the detection system. False negatives may be present in zero or low prevalence reports as primers and/or reaction conditions are critical in the detection system [37, 38]; however, this is unlikely in this study. We employed a highly sensitive methodology (semi-nested PCR) together with a proper positive control, a murine mammary tumor cell line expressing high level MMTV. We estimated the detection limit of this semi-nested PCR method using serially diluted positive control DNA, which was highly sensitive (as little as 80 pg of DNA, not shown). In addition, the primers and PCR conditions used in this study are same as those in previous studies [15–18].

It would be interesting if there was a correlation between HMTV status and clinicopathological parameters. Although several reports demonstrated causal association between these [16, 17, 24, 39], no strong correlation could be found after comprehensive meta-analysis [40]. The HMTV-positive case in this study showed histological grade III with lymph node metastasis. Further studies may reveal some unexplored or unnoticed characteristics of HMTV-associated breast cancers.

## Conclusions

This is the first study to report the prevalence of HMTV in breast cancers in Myanmar. The prevalence of HMTV in Myanmar was consistent with other Asian countries with low or zero prevalence. The frequency of infection and HMTV sequence closely resembled that from Vietnam. It appears that HMTV does not play an important role in breast cancer carcinogenesis in most Asian populations.

## Abbreviations

HMTV: Human mammary tumor virus
MMTV: Mouse mammary tumor virus
PCR: Polymerase chain reaction.

## References

[1] Ferlay J, Soerjomataram I, Dikshit R, Eser S, Mathers C, Rebelo M, Parkin DM, Forman D, Bray F (2015). Cancer incidence and mortality worldwide: sources, methods and major patterns in GLOBOCAN 2012. Int J Cancer.

[2] Torre LA, Bray F, Siegel RL, Ferlay J, Lortet-Tieulent J, Jemal A (2015). Global cancer statistics, 2012. CA Cancer J Clin.

[3] Hortobagyi GN, de la Garza SJ, Pritchard K, Amadori D, Haidinger R, Hudis CA, Khaled H, Liu MC, Martin M, Namer M, O’haughnessy JA, Shen ZZ, Albain KS, ABREAST I (2005). The global breast cancer burden: variations in epidemiology and survival. Clin Breast Cancer.

[4] Jemal A, Center MM, DeSantis C, Ward EM (2010). Global patterns of cancer incidence and mortality rates and trends. Cancer Epidemiol Biomarkers Prev.

[5] Amarante MK, Watanabe MA (2009). The possible involvement of virus in breast cancer. J Cancer Res Clin Oncol.

[6] Lawson JS, Verma M (2009). Do Viruses Cause Breast Cancer?. Cancer Epidemiology.

[7] Callahan R, Smith GH (2000). MMTV-induced mammary tumorigenesis: gene discovery, progression to malignancy and cellular pathways. Oncogene.

[8] Callahan R, Smith GH (2008). Common integration sites for MMTV in viral induced mouse mammary tumors. J Mammary Gland Biol Neoplasia.

[9] Wang Y, Holland JF, Bleiweiss IJ, Melana S, Liu X, Pelisson I, Cantarella A, Stellrecht K, Mani S, Pogo BG (1995). Detection of mammary tumor virus env gene-like sequences in human breast cancer. Cancer Res.

[10] Liu B, Wang Y, Melana SM, Pelisson I, Najfeld V, Holland JF, Pogo BG (2001). Identification of a proviral structure in human breast cancer. Cancer Res.

[11] Nartey T, Mazzanti CM, Melana S, Glenn WK, Bevilacqua G, Holland JF, Whitaker NJ, Lawson JS, Pogo BG (2017). Mouse mammary tumor-like virus (MMTV) is present in human breast tissue before development of virally associated breast cancer. Infect Agent Cancer.

[12] Moore MA (2014). Cancer control programs in East Asia: evidence from the international literature. J Prev Med Public Health.

[13] Lakhani SR, Ellis IO, Schnitt SJ, Tan PH, van de Vijver MJ (2012). WHO Classification of Tumours of the Breast.

[14] Elston CW, Ellis IO (1991). Pathological prognostic factors in breast cancer. I. The value of histological grade in breast cancer: experience from a large study with long-term follow-up. Histopathology.

[15] Ford CE, Tran D, Deng Y, Ta VT, Rawlinson WD, Lawson JS (2003). Mouse mammary tumor virus-like gene sequences in breast tumors of Australian and Vietnamese women. Clin Cancer Res.

[16] Ford CE, Faedo M, Crouch R, Lawson JS, Rawlinson WD (2004). Progression from normal breast pathology to breast cancer is associated with increasing prevalence of mouse mammary tumor virus-like sequences in men and women. Cancer Res.

[17] Hachana M, Trimeche M, Ziadi S, Amara K, Gaddas N, Mokni M, Korbi S (2008). Prevalence and characteristics of the MMTV-like associated breast carcinomas in Tunisia. Cancer Lett.

[18] Mazzanti CM, Al Hamad M, Fanelli G, Scatena C, Zammarchi F, Zavaglia K, Lessi F, Pistello M, Naccarato AG, Bevilacqua G (2011). A mouse mammary tumor virus env-like exogenous sequence is strictly related to progression of human sporadic breast carcinoma. Am J Pathol.

[19] Cedro-Tanda A, Códova-Solis A, Juáez-Cedillo T, Pina-Jiméez E, Hernádez-Caballero ME, Moctezuma-Meza C, Castelazo-Rico G, Góez-Delgado A, Monsalvo-Reyes AC, Salamanca-Góez FA, Arenas-Aranda DJ, Garcí-Hernádez N (2014). Prevalence of HMTV in breast carcinomas and unaffected tissue from Mexican women. BMC Cancer.

[20] Etkind P, Du J, Khan A, Pillitteri J, Wiernik PH (2000). Mouse mammary tumor virus-like ENV gene sequences in human breast tumors and in a lymphoma of a breast cancer patient. Clin Cancer Res.

[21] Glenn WK, Heng B, Delprado W, Iacopetta B, Whitaker NJ, Lawson JS (2012). Epstein-Barr virus, human papillomavirus and mouse mammary tumour virus as multiple viruses in breast cancer. PLoS One.

[22] Levine PH, Pogo BG, Klouj A, Coronel S, Woodson K, Melana SM, Mourali N, Holland JF (2004). Increasing evidence for a human breast carcinoma virus with geographic differences. Cancer.

[23] Melana SM, Picconi MA, Rossi C, Mural J, Alonio LV, Teyssie A, Holland JF, Pogo BG (2002). [Detection of murine mammary tumor virus (MMTV) env gene-like sequences in breast cancer from Argentine patients]. Medicina (B Aires).

[24] Pogo BG, Melana SM, Holland JF, Mandeli JF, Pilotti S, Casalini P, Menard S (1999). Sequences homologous to the mouse mammary tumor virus env gene in human breast carcinoma correlate with overexpression of laminin receptor. Clin Cancer Res.

[25] Slaoui M, El Mzibri M, Razine R, Qmichou Z, Attaleb M, Amrani M (2014). Detection of MMTV-Like sequences in Moroccan breast cancer cases. Infect Agent Cancer.

[26] Bindra A, Muradrasoli S, Kisekka R, Nordgren H, Warnberg F, Blomberg J (2007). Search for DNA of exogenous mouse mammary tumor virus-related virus in human breast cancer samples. J Gen Virol.

[27] Frank O, Verbeke C, Schwarz N, Mayer J, Fabarius A, Hehlmann R, Leib-Mosch C, Seifarth W (2008). Variable transcriptional activity of endogenous retroviruses in human breast cancer. J Virol.

[28] Mant C, Gillett C, D’rrigo C, Cason J (2004). Human murine mammary tumour virus-like agents are genetically distinct from endogenous retroviruses and are not detectable in breast cancer cell lines or biopsies. Virology.

[29] Witt A, Hartmann B, Marton E, Zeillinger R, Schreiber M, Kubista E (2003). The mouse mammary tumor virus-like env gene sequence is not detectable in breast cancer tissue of Austrian patients. Oncol Rep.

[30] Fukuoka H, Moriuchi M, Yano H, Nagayasu T, Moriuchi H (2008). No association of mouse mammary tumor virus-related retrovirus with Japanese cases of breast cancer. J Med Virol.

[31] Ahangar Oskouee M, Shahmahmoodi S, Jalilvand S, Mahmoodi M, Ziaee AA, Esmaeili HA, Mokhtari-Azad T, Yousefi M, Mollaei-Kandelous Y, Nategh R (2014). No evidence of mammary tumor virus env gene-like sequences among Iranian women with breast cancer. Intervirology.

[32] Luo T, Wu XT, Zhang MM, Qian K (2006). [Study of mouse mammary tumor virus-like gene sequences expressing in breast tumors of Chinese women]. Sichuan Da Xue Xue Bao Yi Xue Ban.

[33] Naushad W, Bin Rahat T, Gomez MK, Ashiq MT, Younas M, Sadia H (2014). Detection and identification of mouse mammary tumor virus-like DNA sequences in blood and breast tissues of breast cancer patients. Tumour Biol.

[34] Reza MA, Reza MH, Mahdiyeh L, Mehdi F, Hamid ZN (2015). Evaluation Frequency of Merkel Cell Polyoma, Epstein-Barr and Mouse Mammary Tumor Viruses in Patients with Breast Cancer in Kerman, Southeast of Iran. Asian Pac J Cancer Prev.

[35] Leong SP, Shen ZZ, Liu TJ, Agarwal G, Tajima T, Paik NS, Sandelin K, Derossis A, Cody H, Foulkes WD (2010). Is breast cancer the same disease in Asian and Western countries. World J Surg.

[36] Stewart TH, Sage RD, Stewart AF, Cameron DW (2000). Breast cancer incidence highest in the range of one species of house mouse, Mus domesticus. Br J Cancer.

[37] Glenn WK, Salmons B, Lawson JS, Whitaker NJ (2010). Mouse mammary tumor-like virus and human breast cancer. Breast Cancer Res Treat.

[38] Holland JF, Pogo BG (2012). Comment on the review by Joshi and Buehring. Breast Cancer Res Treat.

[39] Faedo M, Ford CE, Mehta R, Blazek K, Rawlinson WD (2004). Mouse mammary tumor-like virus is associated with p53 nuclear accumulation and progesterone receptor positivity but not estrogen positivity in human female breast cancer. Clin Cancer Res.

[40] Wang F, Hou J, Shen Q, Yue Y, Xie F, Wang X, Jin H (2014). Mouse mammary tumor virus-like virus infection and the risk of human breast cancer. Am J Transl Res.

